# A Cross-Sectional Study on Immune-Inflammatory Responses: Exposure to Polycyclic Aromatic Hydrocarbons Alters Pteridine Metabolism and Activates the Kynurenine Pathway

**DOI:** 10.1021/acs.chemrestox.6c00179

**Published:** 2026-07-06

**Authors:** Terken Baydar, Saziye Sezin Palabiyik-Yucelik, Gözde Girgin, Hande Sipahi, Engin Tutkun, Omer Hinc Yilmaz

**Affiliations:** † Department of Toxicology, Faculty of Pharmacy, 37515 Hacettepe University, Ankara 06800, Turkey; ‡ Department of Toxicology, Faculty of Pharmacy, 37139 Ondokuz Mayıs University, Samsun 55270, Turkey; § Department of Toxicology, Faculty of Pharmacy, 52998 University of Health Sciences, Istanbul 34668, Turkey; ∥ Ankara Occupational Diseases Hospital, Keciören, Ankara 06280, Turkey

## Abstract

Polycyclic aromatic hydrocarbons (PAHs) are common environmental pollutants generated from the incomplete combustion of organic materials and represent an important source of occupational and environmental exposure. Aside from their carcinogenic properties, PAHs are known to exert immunomodulatory and proinflammatory effects. Immune activation is closely associated with alterations in pteridine metabolism and activation of the kynurenine pathway; however, evidence linking PAH exposure to these immune-inflammatory pathways in humans remains limited. This study aimed to investigate the systemic biological effects of occupational exposure to PAHs from asphalt fumes in road construction workers, with a particular focus on alterations in pteridine metabolism and activation of the kynurenine pathway, using a combined panel of exposure and mechanistic biomarkers. Routine clinical parameters remained within normal ranges while urinary 1-hydroxypyrene, a well-established biomarker of internal PAH exposure, was significantly elevated, confirming substantial PAH exposure. Key findings revealed profound alterations in two critical metabolic pathways: (i) Pteridine metabolism was shifted, with increased neopterin and decreased biopterin levels, indicating activation of cell-mediated immunity and reduced cofactor availability; (ii) the kynurenine pathway was concurrently activated, as reflected by elevated kynurenine, reduced tryptophan, and increased estimated indoleamine 2,3-dioxygenase activity. These findings indicate that low-level but chronic PAH exposure induces sustained Th1-type immune activation and metabolic disturbances in the absence of overt clinical pathology, representing a state of subclinical biological adaptation.

## Graphical abstract

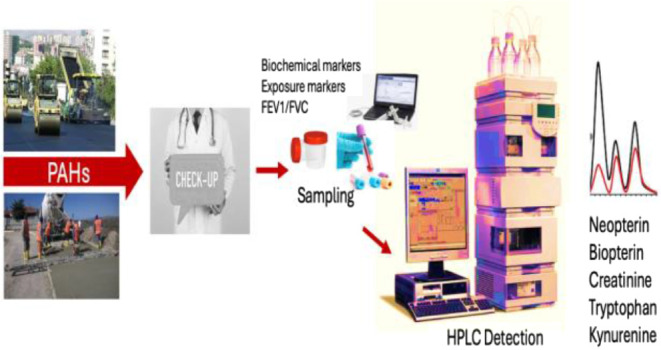

## Introduction

Bitumen is the term more commonly used in Europe to refer to asphalt which is extensively used in road paving, roofing, and waterproofing.^1^ During pavement construction due to elevated temperatures, bitumen fumes release into the air.^2^ These emissions consist of a complex mixture of organic compounds including polycyclic aromatic hydrocarbons (PAHs),^1–3^ volatile organic compounds (VOCs), sulfur, nitrogen oxides, and carbon monoxide.^1,2^ PAHs are a category of complex organic chemicals that are formed during the incomplete combustion of organic materials such as coal, oil, gas, wood, and tobacco. Many of these compounds are known to cause cancer.^1–3^ Asphalt paving workers have been shown to be exposed to PAHs through inhalation and dermal absorption of asphalt vapors and fumes.^1,4,5^ PAHs and VOCs are important classes of environmental xenobiotics that exert complex effects on the immune system, with documented immunomodulatory impacts on both humoral and cell-mediated immunity.^6,7^ Urinary 1-hydroxypyrene (1-OHP), a metabolite of pyrene, is the most commonly used biological marker for assessing occupational exposure to PAHs.^1^ Pyrene is regarded as an effective marker for environmental PAH exposure due to its consistent presence in PAH mixtures and shows strong correlations with airborne pyrene levels and total airborne PAH concentrations.^4,8,9^ However, due to the variable composition of PAH mixtures from different sources, pyrene may not fully represent all exposure conditions.^10^ Toluene and benzene are the VOCs that are most frequently found in asphalt fumes. There is a link between benzene exposure in the workplace and the urinary excretion of phenol and other benzene metabolites.^11^ After exposure to PAHs, most of the chemicals that are absorbed are turned into phenolic metabolites and then eliminated in urine.^12^ Hippuric acid, the primary urinary metabolite of toluene, is also widely recognized as a biological marker for occupational toluene exposure.^13^

Tetrahydrobiopterin (BH_4_) is a necessary cofactor for the hydroxylation of aromatic acids, which is essential for the synthesis of major hormones and neurotransmitters such as catecholamines, serotonin, and melatonin. Its biosynthesis begins with guanosine triphosphate (GTP) which is converted to 7,8-dihydroneopterin by GTP-cyclohydrolase-I (GTP-CH-I). This intermediate is subsequently converted to BH_4_ in the presence of 6-pyruvoyl tetrahydropterin synthase. BH_4_ is readily oxidized to biopterin which is predominantly excreted in urine in either reduced or oxidized forms. Disruption of BH_4_ homeostasis leads to significant alterations in metabolic pathways that underlie the etiology of some pathologies.^14^ Activation of GTP-CH-I by proinflammatory cytokines, particularly interferon-gamma (IFN-γ) also promotes the production of neopterin. Neopterin synthesis is further induced by tumor necrosis factor-alpha (TNF-α), interleukin-2 (IL-2), granulocyte/monocyte colony-stimulating factor, and lipopolysaccharide. Consequently, neopterin is widely used as a sensitive biomarker for the detection and monitoring of T helper 1 (Th1)-type immune activation and inflammatory responses in humans. Previous studies have reported that neopterin levels are elevated, reflecting immune system activation in association with various occupational exposures.^15–20^

It has also been observed that immune-related changes such as tryptophan (TRP) degradation and increased release of neopterin and/or biopterin, are correlated in certain occupational exposures.^16–19^ The essential amino acid TRP is a precursor to two important metabolic pathways: the production of the neurotransmitter serotonin and the formation of kynurenine (KYN) derivatives and nicotinamide adenine dinucleotides.^21^ In the human body, TRP metabolism occurs predominantly via the kynurenine pathway. Tryptophan 2,3-dioxygenase (TDO) in the liver and indoleamine 2,3-dioxygenase (IDO) in extrahepatic tissues catalyze the oxidation of TRP to KYN. Activation of TDO leads to decreased circulating TRP concentrations and increased KYN levels. Consequently, the KYN to TRP ratio (KYN/TRP), representing IDO activity, increases. However, this increase is generally modest and not influenced by immune activation. In contrast, IDO is strongly inducible by proinflammatory cytokines, with interferon-gamma (IFN-γ), produced by activated T cells, being the most potent inducer.^22–25^ To confirm that greater TRP degradation is mediated by IDO rather than TDO, it is essential to exhibit a simultaneous rise in immune system activation markers, such as neopterin.^26^

This study aimed to evaluate potential immunomodulatory effects of occupational exposure to asphalt fumes by assessing alterations in pteridine metabolism with neopterin and biopterin levels, and TRP metabolism by detecting KYN concentrations and IDO activity. To this end, exposure to PAHs and VOCs among bitumen workers was characterized by using established occupational exposure biomarkers, including urinary 1-OHP, hippuric acid, and phenol.

## Materials and Methods

### Study Population and Sampling

The exposed group in the study consisted of 70 males working in road paving operations who presented to the Occupational Diseases Hospital for routine medical control. The median age of the workers was 48 years, and the mean duration of occupational exposure was 17 ± 0.6 months. Information regarding smoking status and dietary habits was not systematically collected as part of this occupational health surveillance study. However, all participants were actively employed and underwent routine medical examinations; participants with active infections or chronic diseases were excluded. Each of the workers was actively working during the study period. Blood and urine samples were collected at the end of the work shift during the summer season, corresponding to the active road paving period.

The control group consisted of 40 healthy male subjects with no history of occupational exposure to PAHs or VOCs. Participants in the control group were selected from those without chronic diseases, active infections, or medication use during the sample collection period. The median age of the control subjects was 34 years. Blood and urine samples from the control group were collected in the early morning hours.

All biological samples were protected from direct light exposure and stored at −20 °C until analysis. The principles of the Local Ethical Committee of the Hospital (#HEK12/10–9) according to the Helsinki Declaration were followed during the whole study.

### Measurements

Routine blood parameters and occupational exposure biomarkers including urinary 1-OHP, hippuric acid, phenol, and catechol (*o*-hydroxyphenol) were analyzed in the hospital biochemistry laboratory using standard bioanalytical procedures. As 24-h urine samples were not collected, biomarker concentrations were normalized to urinary creatinine levels to account for variations in urine dilution and to reduce interindividual variability. Results were therefore expressed as creatinine-adjusted values. Pulmonary function tests were performed for each worker using spirometry. The measured parameters included forced expiratory volume in the first second (FEV_1_), forced vital capacity (FVC), and the FEV_1_/FVC ratio.

High-performance liquid chromatography (HPLC) was used to determine creatinine, neopterin,^27^ and biopterin^28^ levels, and TRP and KYN concentrations^29^ as previously described. Neopterin and biopterin levels in urine samples were quantified using HPLC (HP Agilent 1100 series, Vienna, Austria) with fluorescence detection (HP Agilent 1100 series, Vienna, Austria) with potassium dihydrogen phosphate buffer (pH 7.0) containing 2.5% methanol (v/v) as a mobile phase, and a column (25 cm × 4.6 mm) containing octyl-dodecyl silica gel C18 (5 μm particle size; Hichrom, Berkshire, UK). Measurements of neopterin were taken at a wavelength of 353 nm for excitation (λex) and 438 nm for emission (λem) while biopterin levels were detected at λex, 353 nm and λem, 438 nm. Creatinine determinations were done simultaneously by using an ultraviolet (UV) detector at a wavelength of 235 nm. The neopterin and biopterin levels were expressed as μmol of neopterin/biopterin per mole of creatinine.

The HPLC (HP Agilent 1200 series, Vienna, Austria) was used to detect blood TRP and KYN levels using a reversed-phase ACE C18 column (5 μm, 25 cm × 4.6 mm inner diameter; Avantor, Vienna, Austria). TRP levels were measured by fluorescence detection (λex: 285 nm, λem: 365 nm), while KYN levels were detected simultaneously with UV absorption at 360 nm. The mobile phase was 15 mM potassium dihydrogen phosphate buffer containing 7% acetonitrile (v/v) at pH 6.4. The concentrations of TRP and KYN were both represented in μmole per liter. IDO activity was estimated indirectly by calculating the KYN-to-TRP ratio and was expressed as μmol/mmol.

### Statistical Analysis

Demographic information and data obtained from the measurements were evaluated using SPSS statistical software. The results were presented as mean and standard deviation (SD). The normality of the data was evaluated with Kolmogorov–Smirnov test. The Mann–Whitney *U* test was used to compare two independent groups, and the Spearman nonparametric correlation test was used to detect correlations between the measured parameters. *p* < 0.05 was considered to indicate statistical significance.

## Results

### Exposure Assessment with Routine Clinical Parameters

Consistent with occupational exposure to asphalt fumes, urinary 1-OHP levels were significantly elevated in the worker group, as shown in Table 1. Hippuric acid, catechol, and phenol results and blood biochemical parameters were within normal clinical reference intervals (Table 1).

**Table 1:** Blood Biochemical Findings and Urinary Exposure Markers of the Exposed Workers

Parameter	Bitumen workers	Range
**Biochemical (blood) marker**		
Alanine aminotransferase (ALT), U/L	28.6 ± 0.9	10–40 ^30^
Aspartate aminotransferase (AST), U/L	22.3 ± 0.4	10–30 ^30^
Erythrocyte sedimentation rate (ESR), mm/h	6.35 ± 0.4	≤15 (<50 years, male)^31^
Hematocrit (Hct), %	45.02 ± 0.24	42–50 (male)^30^
Hemoglobin (Hgb), g/dL	15.25 ± 0.80	14–18 (male)^30^
Platelet count (Plt), ×10^3^ cells/mm^3^	241.8 ± 3.5	150–350 ^30^
White blood cell count (WBC), ×10^3^ cells/mm^3^	8.23 ± 0.17	4.5–11.0 ^30^
**Exposure (urinary) marker**		
1-Hydroxypyrene (μmol/mol creatinine)	3.78 ± 0.34	0.24, (nonsmokers); 0.76 (smokers)^32^
Catechol (mg/g creatinine)	1.25 ± 0.98	0.25–4.78 ^33^
Hippuric acid (g/g creatinine)	0.98 ± 0.07	<1 ^34,35^
Phenol (mg/g creatinine)	13.93 ± 1.05	4.5–20.7 ^36^

Based on the spirometry measurements (FVC, FEV_1_, FEV_1_/FVC), the physician-assessed categorization revealed the prevalence of normal, restrictive, and obstructive patterns among the workers (Figure 1).

**Figure 1:**
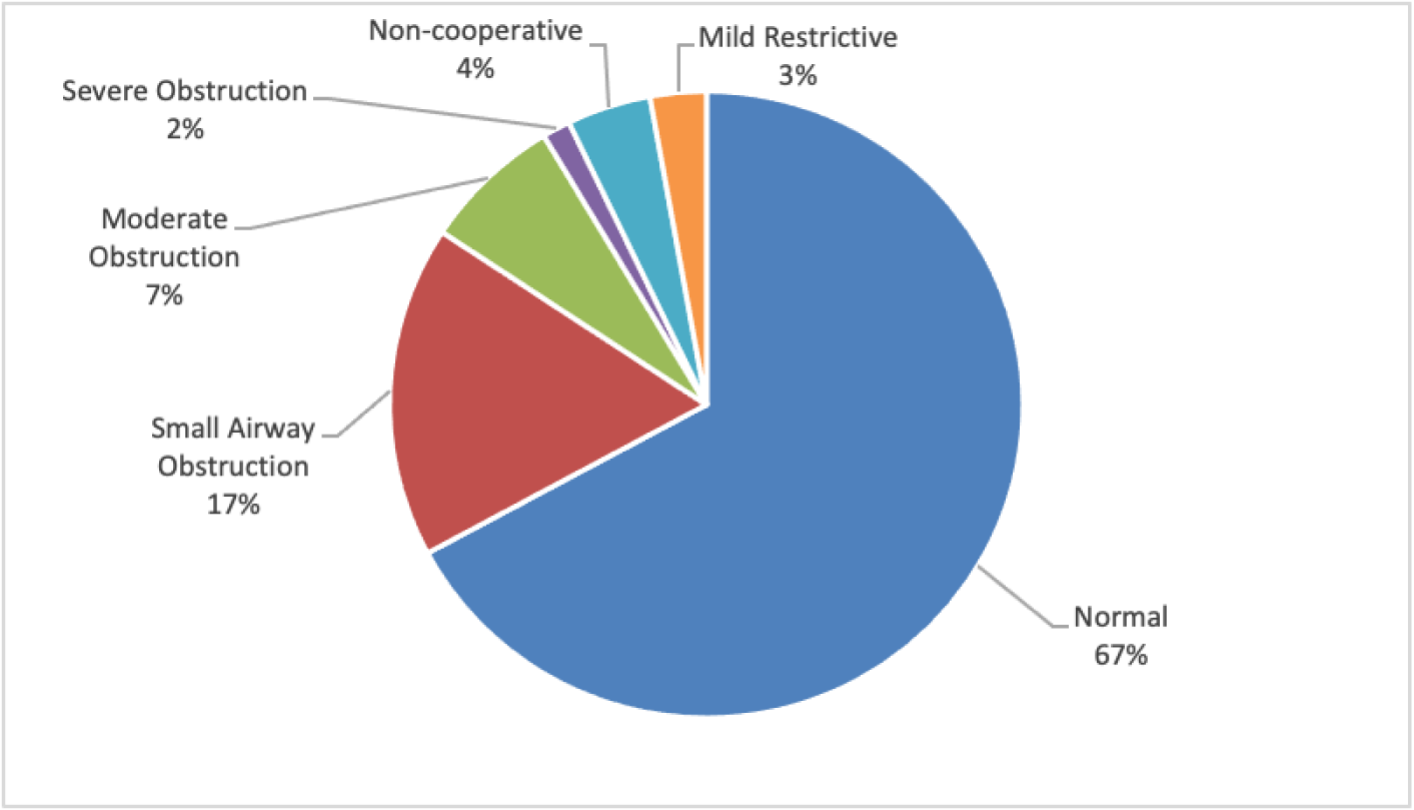
Prevalence of pulmonary function status among bitumen workers.

### Association of Working Duration with Hematological Parameters

To assess long-term exposure effects, correlations between working duration and routine hematological parameters were analyzed. A significant negative correlation was observed between working duration and erythrocyte sedimentation rate (ESR) (Rs= −0.241, *p* = 0.047). In contrast, positive correlations were found between working duration and both hematocrit values (Rs = 0.369, *p* = 0.002) and hemoglobin levels (Rs = 0.338, *p* = 0.004). Work duration was negatively associated with erythrocyte sedimentation rate and positively associated with hematocrit and hemoglobin levels, suggesting long-term physiological adaptation.

### Alterations in Pteridine Metabolism

Urinary pteridine profiles were significantly altered in exposed workers as shown in Figure 2. Neopterin levels were markedly higher in the worker group (188 ± 55 μmol/mol creatinine) compared to the controls (150 ± 48 μmol/mol creatinine) (*p* < 0.01). Conversely, biopterin levels were significantly lower in workers (43 ± 17 μmol/mol creatinine) than in controls (109 ± 124 μmol/mol creatinine) (*p* < 0.01).

**Figure 2:**
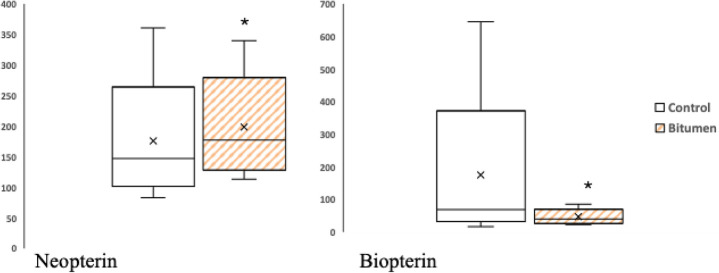
Comparison of urinary neopterin and biopterin levels between exposed workers and nonexposed controls. x, median; *p < 0.01 vs control.

### Activation of the Kynurenine Pathway

The KYN pathway of TRP metabolism showed significant activation in the exposed group as shown in Figure 3. Plasma TRP levels were decreased, while KYN levels were increased in workers compared to controls (both, *p* < 0.01). Consequently, the calculated IDO activity was significantly elevated in the worker group (46.3 ± 10.5 μmol/mmol) relative to the controls (27.8 ± 7.7 μmol/mmol) (*p* < 0.01).

**Figure 3:**
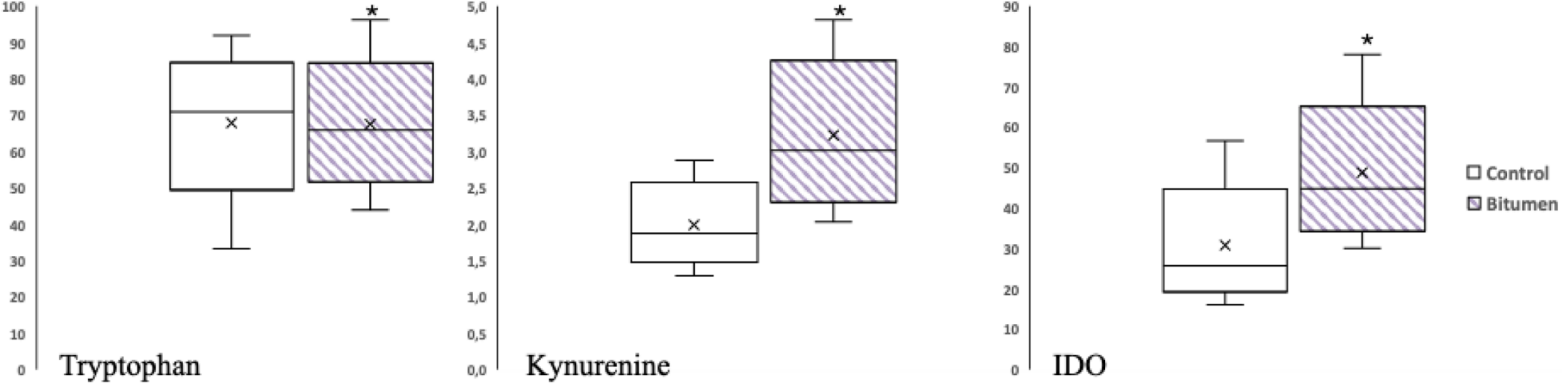
Comparison of blood kynurenine pathway between exposed workers and nonexposed controls. x, median; *p < 0.01 vs control.

### Correlations between Exposure, Functional, and Mechanistic Biomarkers

To explore potential interactions and pathways, Spearman correlation analyses were conducted among key biomarkers. Statistically significant correlations between occupational exposure markers (excluding urinary 1-OHP) and the measured biochemical parameters (neopterin, biopterin, and tryptophan) were observed and summarized in Figure 4. Specifically, significant correlations were identified for phenol, hippuric acid, and catechol with the mechanistic biomarkers, while 1-OHP did not show statistically significant associations with these parameters.

**Figure 4:**
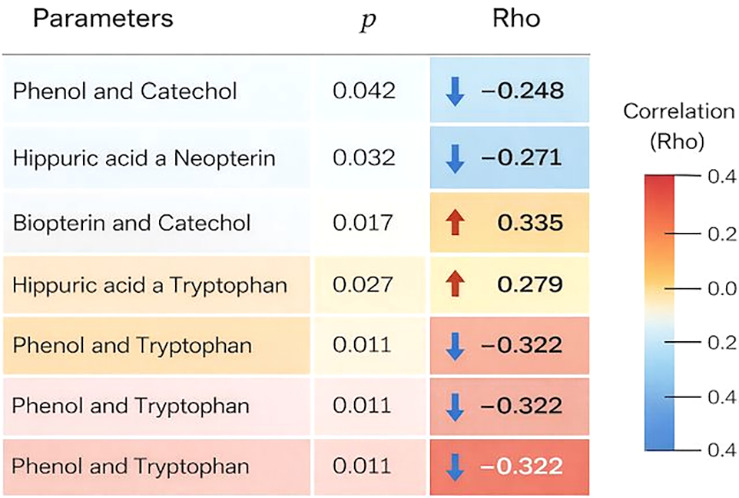
Significant correlations between occupational biomarkers and measured parameters. Rho, Spearman’s correlation coefficient; *p*, Statistical significance.

Significant correlations between exposure biomarkers (e.g., phenol, hippuric acid) and mechanistic biomarkers (neopterin, biopterin, tryptophan) established a direct link between PAH exposure and systemic biological effects.

## Discussion

The present findings indicate that although routine clinical and hematological parameters largely remained within reference ranges, significant alterations were observed in specific metabolic pathways related to immune modulation and oxidative stress. These subtle yet biologically consistent changes suggest the presence of a chronic, low-grade biological perturbation associated with long-term occupational exposure. It should be noted that the exposed worker group had a higher median age compared to the control group. Age-related changes in immune function, including immunosenescence and chronic low-grade inflammation (inflammaging), have been associated with alterations in neopterin levels and tryptophan metabolism.^26,37–39^ However, the neopterin levels observed in our worker group (188 ± 55 μmol/mol creatinine) substantially exceeded typical age-related increases reported in healthy aging populations.^14,26^ Furthermore, the observed correlation patterns and the magnitude of pathway activation are consistent with exposure-related effects rather than age-driven changes alone. Nevertheless, we acknowledge that the age disparity represents a limitation of this study, and future research should employ age-matched control groups to more precisely isolate the effects of occupational PAH exposure.

### Exposure Confirmation and Baseline Health Status

The significantly elevated urinary 1-OHP levels observed in the worker group confirm a substantial absorption of PAHs, which are major constituents of bitumen fumes.^1–3,5^ This finding validates the primary exposure condition of the study. In contrast, the finding that hippuric acid, phenol, and standard blood biochemical parameters (e.g., hepatic enzymes, renal function markers) remained within clinically accepted reference ranges. This observation is consistent with previous occupational studies reporting that chronic bitumen exposure often does not produce overt clinical pathology or marked alterations in routine blood tests.^40^ These findings highlight the limitations of relying solely on standard clinical chemistry for early detection of toxicant-induced stress. The preservation of these parameters may reflect effective homeostatic compensation or the subclinical nature of exposure related effects, highlighting the need for more sensitive, mechanism-based biomarkers.

The absence of significant correlations between urinary 1-OHP and the mechanistic biomarkers is noteworthy. This finding may reflect several factors: first, 1-OHP is a biomarker of recent (24–48 h) PAH exposure, whereas the immune-metabolic alterations observed in this study likely represent cumulative effects of chronic exposure. Second, PAH mixtures in asphalt fumes contain numerous compounds, and 1-OHP may not fully capture the complexity of exposure that drives biological responses. Third, the biological effects on pteridine and kynurenine pathways may be more strongly influenced by other components of asphalt fumes, such as VOCs, for which hippuric acid and phenol serve as biomarkers. This interpretation is supported by the significant correlations observed between these latter biomarkers and the mechanistic parameters. Collectively, these findings underscore the value of using multiple biomarkers to characterize both exposure and biological response in occupational studies.

### Hematological Adaptations and Inflammatory Responses

The observed hematological correlations suggest a complex pattern of biological adaptation. The positive correlation between duration of occupational exposure and both hematocrit and hemoglobin levels is noteworthy. Although this finding may indicate a compensatory response to potential chronic hypoxic stress or sustained occupational burden, it should be interpreted with caution. It may also indicate a type of secondary polycythemia, possibly linked to chronic inflammatory conditions or subtle renal responses; however, additional research is required to validate this hypothesis. More compelling is the significant negative correlation between working duration and ESR which is a nonspecific marker of acute and chronic inflammation. The observed decline in ESR over time may reflect an adaptive attenuation of the acute-phase response or a shift in inflammatory dynamics. Rather than indicating a simple reduction in inflammatory activity, this pattern may suggest a shift in immune function toward a distinct immunoregulatory phenotype.^41–43^

### Pteridine Metabolism: A Marker of Cellular Immune Activation

The significant alterations observed in urinary pteridine levels provide strong evidence of immune system involvement in workers exposed to asphalt fumes. In particular, the elevated neopterin levels indicate activation of cell-mediated immunity, most likely reflecting a Th1-type immune response. Neopterin is a sensitive biomarker of cellular immune activation and is produced by macrophages upon stimulation with IFN-γ. On the other hand, decreased biopterin levels, observed in the context of BH_4_ depletion, may link oxidative stress and nitric oxide synthase uncoupling.^14,26,44^ The marked increase in neopterin levels among exposed workers therefore suggests persistent activation of the cellular immune system, which is commonly associated with chronic oxidative stress and exposure to immune-toxic environmental agents. Conversely, the decrease in biopterin is also of considerable biological relevance. Biopterin is a cofactor for several critical enzymes, including those involved in neurotransmitter synthesis and nitric oxide production. Reduced biopterin bioavailability can impair these pathways, contributing to endothelial dysfunction and increased oxidative stress.^45^ Notably, the inverse relationship between neopterin and biopterin observed in this study represents a well-recognized metabolic pattern associated with immune activation. Under inflammatory and oxidative conditions, the metabolic flux within the pteridine pathway tends to shift toward enhanced neopterin production at the expense of biopterin synthesis.^46^ This metabolic redistribution can be interpreted as a quantifiable biochemical signature of sustained immune activation and biological stress.

The substantially larger variance in urinary biopterin levels observed in the control group compared to the worker group warrants comment. Control participants exhibited a wide range of biopterin values (14–644 μmol/mol creatinine), with a SD of 124 μmol/mol creatinine, whereas workers demonstrated a narrower range (22–85.5 μmol/mol creatinine) with a SD of 17 μmol/mol creatinine. This disparity likely reflects the well-documented interindividual variability in biopterin excretion among healthy populations, which can be influenced by factors including genetic polymorphisms in GTP-cyclohydrolase-I, dietary variations, physical activity levels, and circadian rhythms.^47–50^ Conversely, the relative homogeneity of biopterin values in the exposed group suggests that chronic occupational PAH exposure may exert a homogenizing influence on pteridine metabolism, potentially through persistent immune activation that constrains the normal physiological range of biopterin excretion. Importantly, despite the large variance in the control group, the statistically significant difference between groups remains robust (*p* < 0.01), and the median comparison, which is less sensitive to outliers, further supports the finding of decreased biopterin in exposed workers. Future studies with larger sample sizes could employ stratified analyses to better characterize the determinants of interindividual variability in pteridine metabolism.

### Kynurenine Pathway: Implications for Immune Tolerance

During Th1-type immune responses, activated T cells release substantial amounts of proinflammatory cytokines, particularly IL-2 and IFN-γ, which play a crucial role in the development and regulation of cell-mediated immunity. Among other pathways, T cell–derived IFN-γ induces activation of the IDO in macrophages, which converts TRP to *N*-formyl-kynurenine and subsequently to KYN. Activation of the kynurenine pathway therefore represents a key biochemical consequence of inflammatory signaling, linking immune activation to broader metabolic and regulatory processes.

The activation of the kynurenine pathway, evidenced by decreased plasma TRP and increased KYN levels, is one of the most significant findings of this study. IDO is induced by proinflammatory cytokines, particularly IFN-γ, and its activation serves as a bridge between immune activation and systemic physiological responses.^21–25,51^ The depletion of TRP may contribute to the suppression of T-cell proliferation, thereby promoting immune tolerance and immune regulation. This mechanism could partly explain the attenuated acute inflammatory response suggested by the observed decrease in ESR. Simultaneously, the accumulation of KYN and its downstream metabolites can have neuroactive and pro-oxidant properties. This pathway has been implicated in neurotoxicity, fatigue, and depression in various pathological conditions and could underlie some nonspecific neurological symptoms reported by workers in comparable occupational environments.^21,24,52^ The concurrent activation of the neopterin and kynurenine pathways, both driven by IFN-γ, presents a coherent biochemical signature of Th1-mediated immune activation in workers exposed to asphalt fumes.

### A Possible Network of Metabolic Shifts

PAHs are primarily absorbed through inhalation, ingestion, and dermal contact, after which they are metabolized into hydroxylated PAH derivatives and subsequently excreted in urine. Numerous studies have demonstrated a significant relationship between environmental PAH exposure levels and urinary concentrations of PAH metabolites.^8–12^ Therefore, the urine metabolite concentration of PAHs is usually used to detect and evaluate the actual exposure level of PAHs in the environment.^53,54^ In the present study, elevated 1-OHP, a well-established biomarker of PAH exposure, was detected in workers exposed to asphalt fumes, confirming substantial internal exposure. Urinary PAH metabolites are commonly used as biomarkers to evaluate recent exposure to these compounds. Besides the typical advantages of urinary biomarkers such as being noninvasive and easily obtainable, these metabolites are particularly valuable when exposure occurs through multiple pathways, when direct measurement of external exposure is challenging, or when collecting blood samples is not feasible or acceptable. Individuals who have experienced considerable exposure to PAHs should undergo periodic health assessments, even in the absence of symptoms, to enable the early identification and management of potential PAH-related disorders.^55^

While smoking is a known source of PAH exposure and can influence both 1-OHP levels and immune parameters, the occupational exposure in this study represents a substantial additional burden beyond environmental sources.^56,57^ Dietary factors, particularly TRP intake, could also influence baseline measurements; however, the group comparisons and within-group consistency suggest that the observed differences are primarily driven by occupational exposure rather than dietary variation. Several potential confounding factors warrant consideration. First, the cross-sectional design prevents the establishment of causal relationships, as the reviewer noted. Second, smoking status, which was not systematically recorded, represents a potential confounder, as smoking contributes to PAH exposure and can independently influence 1-OHP levels and immune parameters. However, as shown in Table 1, the substantially elevated 1-OHP levels in the worker group (3.78 ± 0.34 μmol/mol creatinine) compared to established reference values for smokers (0.76 μmol/mol creatinine) indicate that occupational exposure was the dominant source of PAH exposure.^32^ Third, dietary habits and nutritional status could influence tryptophan metabolism and pteridine levels; however, the consistent patterns observed across the worker group suggest that these factors did not significantly confound the overall findings. Fourth, coexposure to other occupational agents (e.g., silica, heavy metals) present in road construction environments could contribute to the observed immune alterations. While the primary exposure of interest was asphalt fumes, future studies should incorporate comprehensive exposure assessments to disentangle the effects of individual agents.

The significant correlations between exposure biomarkers (phenol, catechol, hippuric acid) and the mechanistic biomarkers (neopterin, biopterin, TRP) provide important information about how external exposure translates into internal biological responses. These associations indicate that occupational exposure to aromatic hydrocarbons may influence specific metabolic pathways associated with immune activation and oxidative stress. For example, the negative correlation between hippuric acid and neopterin and the positive correlation between catechol and biopterin suggest that different exposure components or their metabolites may differentially modulate pteridine metabolism. Moreover, the inverse correlation between phenol and TRP levels supports a possible association between aromatic compound exposure and increased TRP catabolism via the KYN pathway.

In summary, our results indicate that although the workers did not exhibit overt clinical illness, their biochemical profiles revealed a consistent pattern characterized by immune system activation (elevated neopterin levels and increased IDO activity), accompanied by adaptive metabolic alterations, including reduced TRP concentrations and changes in pteridine balance, as well as potential long-term hematological adaptations. This pattern is consistent with chronic, systemic, low-level exposure to complex mixtures containing PAHs and other volatile compounds, in which oxidative stress and inflammatory signaling appear to act as primary mediators.

## Conclusion

By using a functional biomarker approach, the present study has shown that long-term exposure to bitumen fumes at work alters important metabolic pathways that control the immune response and oxidative stress homeostasis. These changes on the pteridine and kynurenine pathways, even when there are no substantial alterations in routine clinical parameters, show their sensitivity as early effect biomarkers. The observed biological profile indicates a condition of chronic, low-grade immune activation, which may have long-term health risks, including immune dysregulation and neuroendocrine effects. The results support the use of sensitive, mechanism-based biomarkers such as neopterin and kynurenine pathway indicators for the early detection of biological effects in occupational health surveillance of complex chemical mixtures.

## Limitations

The cross-sectional design of the study prevents the determination of causal relationships. Additionally, potential confounding factors—including smoking status, dietary habits, and coexposures to other occupational agents—were not fully assessed, and the age disparity between groups represents a notable limitation. The KYN and pteridine findings cannot be fully understood in a clinical setting because there is not enough information about specific neurobehavioral symptoms or more detailed immunophenotyping. Subsequent longitudinal studies should incorporate these biomarkers alongside neuropsychological assessments, comprehensive cytokine profiles, and genetic polymorphisms in metabolic enzymes (e.g., IDO, GTP-CH-I). Furthermore, assessing the potential reversibility of these changes during nonexposure periods would be valuable for risk assessment.

## ⊥

HLC-LAB Consultancy, Çankaya, Ankara, Turkey

## #

Department of Public Health, Faculty of Medicine, Yildirim Beyazit University, Ankara, Turkey.

## Abbreviations

1-OHP: 1-hydroxypyrene
BH_4_: tetrahydrobiopterin
GTP: guanosine triphosphate
GTP-CH-I: GTP-cyclohydrolase-I
FEV: forced expiration volume
FVC: forced vital capacity
HPLC: high-performance liquid chromatography
IDO: indoleamine 2,3-dioxygenase
IL: interleukin
IFN-γ: interferon-gamma
KYN: kynurenine
PAH: polycyclic aromatic hydrocarbon
TDO: tryptophan 2,3-dioxygenase
Th: T helper
TNF-α: tumor necrosis factor-alpha
TRP: tryptophan
VOC: volatile organic compound
λex: lambda-ex (molecular fluorescence excitation wavelength)
λem: lambda-em (molecular fluorescence emission wavelength)
